# Memantine Differentially Regulates Tau Phosphorylation Induced by Chronic Restraint Stress of Varying Duration in Mice

**DOI:** 10.1155/2019/4168472

**Published:** 2019-02-14

**Authors:** Yunsheng Liu, Lan Cao, Xiaoxu Zhang, Yan Liang, Yuxia Xu, Cuiqing Zhu

**Affiliations:** State Key Laboratory of Medical Neurobiology & Institutes of Brain Science, Shanghai Medical College, Fudan University, Shanghai 200032, China

## Abstract

Exposure to chronic psychiatric stress has been linked to Alzheimer's disease-related tau hyperphosphorylation and abnormalities in glutamate neurotransmission. However, the pathological relationship between glutamatergic dysfunction and tau phosphorylation in the cerebral cortex under chronic psychiatric stress is not fully understood. The present study investigated the effects of memantine (MEM, 5 and 10 mg/kg), an uncompetitive N-methyl-D-aspartate (NMDA) receptor antagonist, on chronic restraint stress- (CRS-) induced tau phosphorylation in mice. CRS administered for 16 or 28 consecutive days (1 h daily) induced significant tau phosphorylation in the brain. MEM treatment suppressed the elevation of phosphorylated tau (P-tau) levels induced by 16-day CRS in a dose-dependent manner. P-tau reduction was accompanied by the attenuation of the upregulation of GSK3*β* and CDK5 expression and the downregulation of PP2A activity induced by CRS. Additionally, MEM reduced CRS-induced upregulation of NMDA receptor subunit levels (GluN2A, GluN2B) in the frontal cortex. However, MEM markedly enhanced tau phosphorylation in the frontal cortex and other cerebral cortical regions following 28 days of CRS. The stimulatory effect of MEM on CRS-induced tau phosphorylation was correlated with increased activities of AKT, JNK, and GSK3*β*, inactivation of PP2A, and downregulation of Pin1 and HSP70. Moreover, MEM did not effectively reverse the NMDA receptor upregulation induced by 28-day CRS and even increased GluN2B subunit levels. In contrast to the duration-dependent effects of MEM on P-tau levels, MEM produced an anxiolytic effect in both regimens as revealed by elevated plus maze testing. However, MEM did not affect the body weight reduction induced by CRS. Thus, MEM exerts distinctive effects on CRS-induced tau phosphorylation, which might be related to the expression of GluN2B. The differential effects of MEM on P-tau levels have crucial implications for its clinical application.

## 1. Introduction

Alzheimer's disease (AD) is a neurodegenerative brain disease and the most common cause of dementia. AD is pathologically characterized by the extracellular deposition of *β*-amyloid (A*β*) plaques and the intracellular neurofibrillary tangles (NFTs) composed of aberrantly phosphorylated tau [1]. Since the discoveries of A*β* and tau, numerous studies have addressed the molecular events underlying AD pathogenesis. However, the causes of AD remain controversial, and no effective treatments are available [2].

Multiple factors are involved in the pathogenesis of AD, including aging, sex, endocrine levels, social environment, lifestyle, and stress factors [3]. Among these factors, chronic stress not only induces anxiety-like behavior [4] but also has long been thought to promote the onset of AD and associated brain damage [5–7]. AD is often accompanied by anxiety, with anxious behaviors present in 25–75% of patients with AD [8–12]. In-depth analyses of the occurrence and development mechanism of stress-associated AD may therefore provide a theoretical foundation for the development of effective interventions.

Tau proteins are widely expressed in the central nervous system and play a crucial role in neuronal physiology [13]. In pathological conditions, including stress, tau is abnormally modified, particularly via phosphorylation [14]. Tau hyperphosphorylation induces a conformational change, which contributes to tau dysfunction and promotes the formation of insoluble paired helical filaments (PHFs), the main component of NFTs [15, 16]. Importantly, the relationship between stress and tau pathology has been documented not only in the tau mutant animals but also in wild-type animals [17–22].

Chronic glutamate excitotoxicity has been hypothesized to play a role in AD [23, 24]. Stress increases extracellular glutamate levels [25, 26], while glutamic N-methyl-D-aspartate receptor (NMDAR) antagonists modify the hippocampal synaptic plasticity in both acute and repeated restraint stresses in rats [27]. In addition, memantine (MEM), a low- to moderate-affinity uncompetitive NMDA receptor (NMDAR) antagonist, was reported to reduce anxiety-like behavior in animal models of anxiety [28]. These findings not only suggest a possible role of glutamate in the mechanisms underlying the molecular and cellular alterations in brain caused by stress but also imply that the regulation of glutamatergic function might attenuate the stress-induced pathological changes. However, whether the NMDAR antagonist MEM may decrease stress-induced tau phosphorylation has not been investigated.

MEM has been used to treat moderate to severe AD [29]. MEM was able to protect neurons from A*β* toxicity and alleviated tau hyperphosphorylation in an AD animal model [30, 31]. The NMDARs have also been implicated in the regulation of tau phosphorylation [32]. Therefore, in this study, we tested whether MEM could affect tau phosphorylation induced by chronic restraint stress (CRS) in mice, which simulates everyday emotional stress in humans, and explored the underlying mechanisms. We investigated the expression levels of NMDAR subunits, protein kinases, and phosphatase 2A (PP2A) involved in tau phosphorylation and their active or inactive forms and molecular chaperones. We also examined if MEM had an effect on anxiety-like behaviors induced by CRS.

## 2. Materials and Methods

### 2.1. Antibodies and Reagents

The following primary antibodies were used to visualize tau proteins: AT8 (MN1020, Thermo Scientific, USA), PS396 (44752G, Invitrogen, USA), anti-Tau 3-repeat isoform RD3 (05-803, Millipore, USA), anti-Tau 4-repeat isoform RD4 (05-804, Millipore), and TAU5 (MA5-12805, Invitrogen). To visualize NMDARs, antibodies against GluN2A (PA5-35377, Thermo Scientific) and GluN2B (ab65783, Abcam, USA) were used. To visualize kinases, primary antibodies against cyclin-dependent kinase 5 (CDK5) (Sc-6247, Santa Cruz, USA), glycogen synthase kinase 3 beta (GSK3*β* and p-GSK3*β* (Ser9)) (ab32391 and ab75814, Abcam) protein kinase B (AKT and p-AKT) (#9272 and #4060, Cell Signaling Technology, USA), c-Jun N-terminal kinase (JNK and p-JNK) (#9252 and #4668, Cell Signaling Technology) extracellular signal-regulated kinases (ERK and p-ERK) (#9102 and #9101, Cell Signaling Technology), and P38 mitogen-activated protein kinases (P38MAPK and p-P38MAPK) (#9212 and 9211S, Cell Signaling Technology) were used. Also, antibodies against peptidyl-prolyl *cis*-*trans* isomerase NIMA-interacting-1 (Pin1) (#3722S, Cell Signaling Technology), heat shock proteins 90 (HSP90) (#4874S, Cell Signaling Technology), HSP70 (#4873, Cell Signaling Technology), and HSC70 (AF5183, Affbiotech, USA), as well as against protein phosphatases pY307-PP2Ac and PP2Ac (BS4867 and BS1586, Bioworld Technology, USA), demethylated protein phosphatase PP2A catalytic subunit (DM-PP2Ac) (05-577, Millipore), *β*-actin, and glyceraldehyde 3-phosphate dehydrogenase (GAPDH) (Sc47778 and Sc-25778, Santa Cruz) were used. MEM hydrochloride was purchased from Sigma-Aldrich (M9292, St. Louis, MO, USA) and was dissolved in 0.9% saline.

### 2.2. Animals

Ninety-six male C57BL/6J mice (10-11 weeks old), weighing 24-27 g, were obtained from the Shanghai Experimental Animal Center of the Chinese Academy of Science (Shanghai, China). Mice were maintained at a constant temperature (22–24°C) and humidity (40–60%) under a 12-h light/dark cycle with food and water ad libitum. All procedures were performed strictly in accordance with the National Institutes of Health Guide for the Care and Use of Laboratory Animals and were approved by the Animal Care and Use Committee of the School of Basic Medical Sciences of Fudan University. Every effort was made to minimize animal suffering or discomfort and to reduce the number of animals used.

### 2.3. Experimental Design and Drug Treatment

Experimental animals were randomly divided into 4 groups (10 per group): (1) control (no stress treatment and intraperitoneal (i.p.) injection of isotonic saline), (2) CRS (chronic restraint stress and i.p. saline), (3) CRS + an i.p. injection of 5 mg/kg MEM, and (4) CRS + an i.p. injection of 10 mg/kg MEM. Groups 1 and 2 received identical volume of vehicle (isotonic saline; 30 min before exposure to stress). Groups 3 and 4 received the same volume of MEM containing two different doses (5 mg/kg or 10 mg/kg) 30 min before exposure to stress. The MEM dosages used in this study were based on the previous report [33]. To evaluate the effects of MEM on tau in mice without CRS, animals were also randomly divided into 3 groups (4 per group): (1) control (i.p. injection of isotonic saline), (2) i.p. injection of 5 mg/kg MEM, and (3) i.p. injection of 10 mg/kg MEM. The varying duration for experimental animals and control animals was 16 and 28 consecutive days.

### 2.4. Chronic Restraint Stress

The chronic restraint stress (CRS) method was modified according to procedures described previously [34]. Repeated stress involved placing mice in 50 ml conical tubes with holes for ventilation, without physical pressure or pain, for 1 h per day for 16 or 28 consecutive days. The stress environment was a room with the same temperature, illumination, and background noise as the animal housing room. Nonstressed control mice were cut off food and water in the corresponding CRS time and were transported to/from the testing rooms but were not otherwise handled.

### 2.5. Body Weight and the Elevated Plus Maze (EPM) Test

Body weight was measured every 7 days. The protocol for the EPM test was described previously [35]. The EPM apparatus (MED-VPM-MS, Med Associates, USA) consisted of a central part (6.5 × 6.5 cm), two opposing closed and open arms (35 × 6.5 cm), and nontransparent walls (19 cm in height). To begin the test, an animal was placed in the center of the apparatus facing an open arm. The test session was 5 min in duration. The maze was cleaned with 75% ethanol and completely dried before each test. The numbers of open- and closed-arm entries and the time spent in the open and closed arms were measured. The percentage of time in the open (closed) arms was defined as the time spent in the open (closed) arms divided by the session duration (5 min).

### 2.6. Western Blot Analysis

Mice (4 per group) were anesthetized with sodium pentobarbital (40 mg/kg), which has been demonstrated not to influence tau phosphorylation over the time frame used in this study [36]. After sedation, animals were decapitated and the frontal cortex, hippocampus, and other subregions of the cerebral cortex were rapidly dissected and frozen on dry ice. The samples were homogenized in ice-cold radioimmunoprecipitation assay (RIPA) lysis buffer containing Tris-HCl (20 mM, pH 7.4), NaCl (170 mM), MgCl_2_ (1.5 mM), Na_3_VO_4_ (1 mM), NaF (10 mM), sodium deoxycholate (0.5 mM), EDTA (1 mM), EGTA (0.5 mM), NP40 (1%), and protease and phosphatase inhibitor cocktails (Roche). For the detection of NMDAR subunits (GluN2A, GluN2B), the samples were homogenized in ice-cold lysis buffer containing SDS (2%), Tris-HCl (10 mM, pH 7.4), Na_3_VO_4_ (0.1 mM), NaF (1 mM), and protease and phosphatase inhibitor cocktails [37]. Homogenates were centrifuged at 12,000 ×g for 15 min at 4°C, and the supernatant was collected. Protein concentrations were determined by a Bicinchoninic Acid (BCA) Protein Assay Kit (Biomiga, PW0104) with bovine serum albumin (BSA) (Sigma, USA) as a standard. Supernatant samples were added into same volume of 2x SDS loading buffer and then were heated to 100°C for 5 min. A portion (20 *μ*g) of each sample was separated by 10% SDS-polyacrylamide gel electrophoresis (SDS-PAGE), and then electrophoretically transferred to nitrocellulose membranes (Millipore, USA). The membrane was blocked with 5% nonfat skim milk in TBST (150 mM NaCl, 20 mM Tris-HCl, and 0.1% Tween 20 at pH 7.4) for 1 hour at room temperature and then incubated with the primary antibody in blocking buffer overnight at 4°C. After washing with TBST for 3 times, infrared fluorescent dye- (IRDye-) labeled secondary antibodies (LI-COR, USA) were applied for 1 h at room temperature. After 3 additional washes with TBST buffer, blots were scanned and analyzed by the Odyssey IR Imaging System (LI-COR, USA). Normalization of the results was accomplished by parallel probing for GAPDH or *β*-actin in blots.

### 2.7. Immunofluorescence

In general, frozen samples (brain sections) were washed 3 times with phosphate-buffered saline (PBS) and blocked for 1 hour in PBS containing 0.2% Triton X-100 and 10% normal serum at 37°C. After blocking, sections were washed 3 times with PBS. Immunodetection was performed by incubation with an AT8-specific antibody at the manufacturer-suggested dilution (1 : 200) at 37°C for 1 h, followed by overnight incubation at 4°C. After washing 3 times in PBS, the sections were incubated for 1 h at 37°C with an anti-mouse Alexa Fluor 488-conjugated secondary antibody (1 : 200, Invitrogen). For nuclear labeling, the sections were incubated with 4′,6-diamidino-2-phenylindole (DAPI) for 15 min at 37°C. The sections were washed again 3 times in PBS and then mounted on glass slides with ProLong® Gold Antifade Reagent (Cell Signaling Technology). Images were captured using a Nikon Eclipse Ni-E/Ni-U microscope (Japan).

### 2.8. Statistical Analysis

Statistical analysis of data was carried out by one-way analysis of variance (ANOVA) followed by Tukey's post hoc test for multiple comparisons. Two-way ANOVA followed by Bonferroni's multiple comparison test was used to analyze the change in body weight during the CRS procedure. All statistical analyses were performed using the GraphPad Prism version 6.0 software (GraphPad Software, San Diego, CA, USA). The data are presented as the means ± standard error (SEM). Statistical significance was set at *p* < 0.05.

## 3. Results

### 3.1. Effects of MEM on Body Weight and Behavioral Changes during CRS

**Figure 1:**
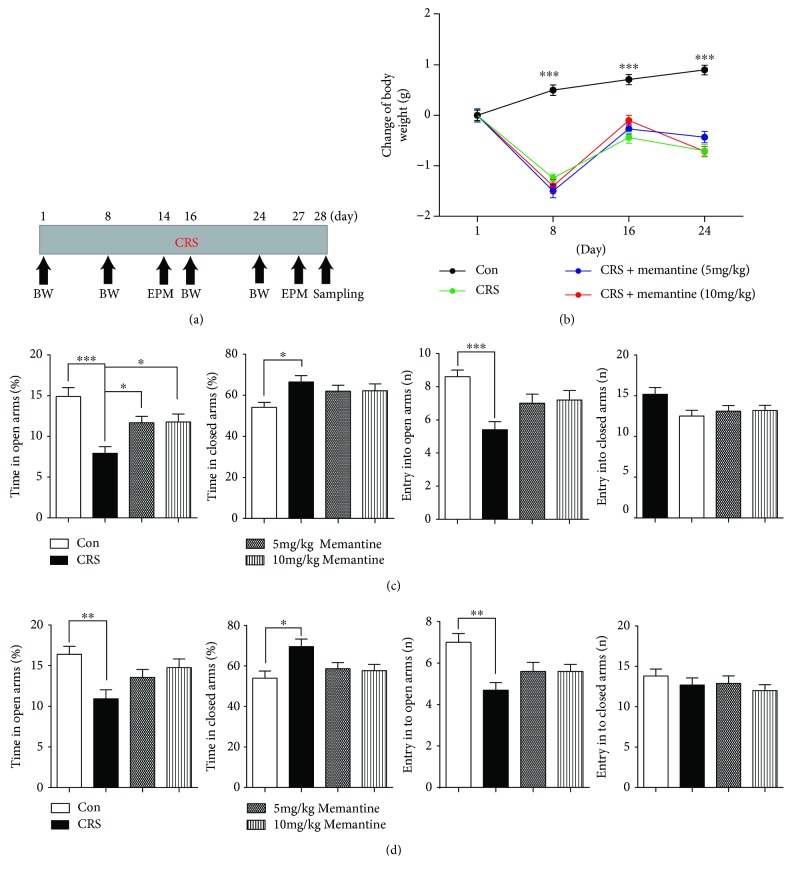
Experimental design, body weight changes, and anxiety testing. (a) Schematic of the chronic restraint stress (CRS) treatment schedule, behavioral testing experiment, and sampling. (b) Body weights in the four groups of mice were measured on days 1, 8, 16, and 24 after CRS commencement. ∗∗∗ represents a comparison between control and CRS mice on days 8, 16, and 24. Memantine intervention had no significant effect on body weight recovery. (c, d) Memantine decreased CRS-induced anxiety in the elevated plus maze test. Time in the open arms, time in the closed arms, and the numbers of open- and closed-arm entries were measured on days 14 (c) and 27 (d). The results are shown as means ± SEM (*n* = 10 in each group). ^∗^*p* < 0.05; ^∗∗^*p* < 0.01; ^∗∗∗^*p* < 0.001. BW: body weight; EPM: elevated plus maze; Con: control; CRS: chronic restraint stress.

A previous study showed that MEM has anxiolytic effects [4]. We explored the effects of MEM on body weight and anxiety levels. A schematic of the consecutive 28-day CRS and MEM treatment regimen, behavioral test, and sampling is shown in Figure 1(a). Mouse body weights were measured on days 1, 8, 16, and 24, and the EPM test was conducted on days 14 and 27.

Results showed that the time effect on body weight was significant during the period of CRS. CRS treatment (*F*_(1, 18)_ = 69.93, *p* < 0.001) also had a significant effect on body weight. In contrast to the gradual increase in body weight in the control group, CRS mice showed a significant weight loss on day 8, partially recovered on day 16, and tended to lose weight again on day 24 (Figure 1(b)). Neither MEM dosage (5 mg/kg, *F*_(1, 18)_ = 0.09118, *p* = 0.7661; 10 mg/kg, *F*_(1, 18)_ = 0.09682, *p* = 0.7593) had a significant effect on body weight at any of the three time points as compared to the CRS group (Figure 1(b)).

In the EPM test (Figures 1(c) and 1(d)), CRS mice spent 46.8% (day 14; *p* < 0.001) and 33.3% (day 27; *p* < 0.01) less time in the open arms compared to the control group. In contrast, the closed-arm residence time in the CRS group was increased by 22.7% (*p* < 0.05) and 28.9% (*p* < 0.05) at the respective time points compared with that of the control mice. Moreover, CRS significantly reduced the number of open-arm entries on day 14 (*p* < 0.001) and day 27 (*p* < 0.01), by 37.2% and 32.9%, respectively, relative to the control group. In contrast, no significant difference was found between the control and CRS groups on the number of closed-arm entries. These results indicated increasing severity of anxiety behavior in mice subjected to CRS commencement.

On day 14, mice treated with 5 or 10 mg/kg MEM showed an increase in the time spent in the open arms (5 mg/kg, *p* < 0.05; 10 mg/kg, *p* < 0.05). Moreover, mice treated with MEM also showed a trend of CRS-induced abnormality reversal in the number of open- and closed-arm entries and in closed-arm residence time. The differences in these indexes were not significant not only between the CRS-only and CRS + MEM groups (*P* > 0.05) but also between the control and CRS + MEM groups (Figure 1(c)). Thus, MEM treatment rendered the CRS-induced changes in EPM test performance statistically nonsignificant. On day 27, mice treated with 5 or 10 mg/kg MEM also showed a nonsignificant trend of CRS-induced behavioral abnormality reversal compared to CRS-only mice, indicating a beneficial effect of MEM on EPM test performance in mice subjected to CRS (Figure 1(d)).

### 3.2. MEM Reduces Tau Hyperphosphorylation Induced by 16-Day CRS

**Figure 2:**
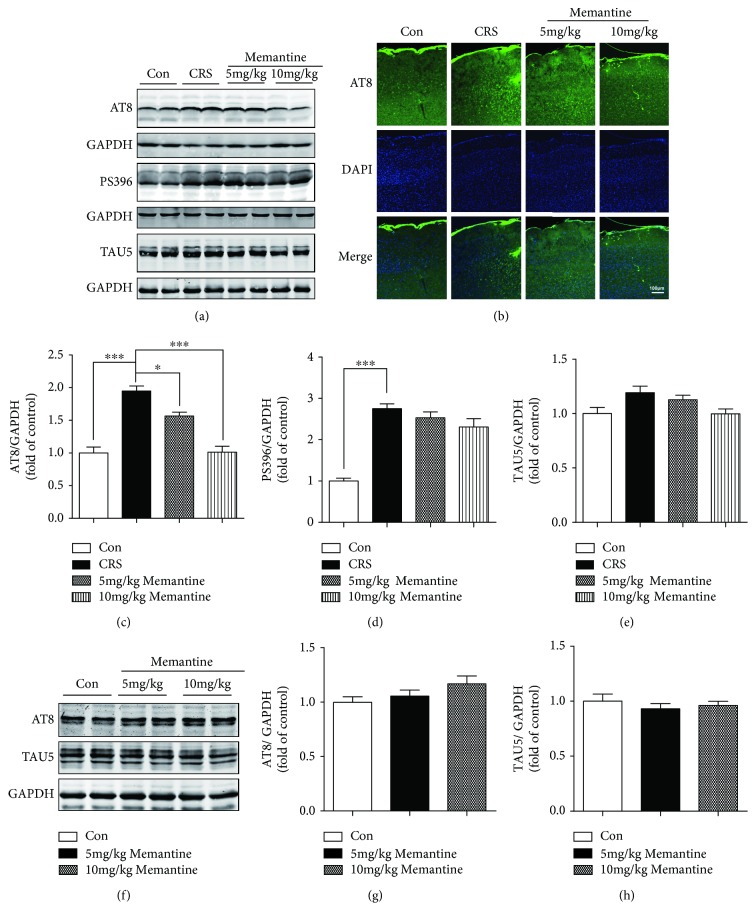
Memantine reduces tau hyperphosphorylation induced by 16 consecutive days of chronic restraint stress (CRS) in the mouse frontal cortex. (a) Frontal cortex extracts were analyzed by Western blotting with the AT8 and PS396 antibodies. Memantine reduced CRS-induced tau phosphorylation at the AT8 site, but not at the Ser396 site. CRS with or without memantine treatment had no statistically significant effect on the expression of total tau (TAU5). (b) Representative areas of AT8-positive immunofluorescent staining in cortices from the four groups of mice. (c–e) Densitometric quantification of the bands in (a). (f) Mice without CRS were treated with memantine in doses of 5 and 10 mg/kg for 16 days; tau phosphorylation and expression were analyzed by Western blotting. (g, h) Densitometric quantification of the bands in (f). The results are shown as means ± SEM (*n* = 4 in each group). GAPDH was used as a loading control. ^∗^*p* < 0.05; ^∗∗∗^*p* < 0.001. Con: control.

Consistent with our previous study showing an increase in phosphorylated tau (P-tau) in the cerebral cortex induced by daily 30-min restraint stress for 18 consecutive days [22], immunoblotting with an AT8 antibody, which detects tau phosphorylated on S199/S202/T205, revealed enhanced tau phosphorylation in the frontal cortex of mice subjected to 1-h restraint stress daily for 16 days (Figure 2(a)). A previous study reported that MK-801, an open-channel blocker that noncompetitively inhibits NMDARs, reduced tau phosphorylation induced by noise exposure for 14 consecutive days [38]. Therefore, we examined the effects of MEM on tau phosphorylation induced in the frontal cortex by 16-day CRS. We found that MEM reduced CRS-induced tau phosphorylation at the AT8 epitope in the frontal cortex of mice subjected to 16-day CRS (Figures 2(a) and 2(c)). The reduction appeared to be dose-dependent, as 5 mg/kg MEM caused a 20% decline in AT8 site phosphorylation (*p* < 0.05, Figures 2(a) and 2(c)) whereas 10 mg/kg MEM induced a 48.2% decrease (*p* < 0.001, Figures 2(a) and 2(c)). Immunofluorescent staining confirmed that 16-day CRS resulted in an upregulation of AT8 P-tau immunoreactivity and MEM treatment minimized this increase (Figure 2(b)). Consistent with the previous research, CRS also significantly increased tau phosphorylation at Ser396 (*p* < 0.001, Figures 2(a) and 2(d)). However, treatment with 10 mg/kg MEM produced a nonsignificant reduction in this phosphorylation. In addition, western blotting with TAU5 antibody, which recognizes both phosphorylated and unphosphorylated forms of tau, showed no significant differences among the control, CRS-only, and CRS + MEM groups (Figures 2(a) and 2(e)). However, CRS caused a trend-level increase in total tau expression, which tended to be reversed by MEM treatment. To reveal whether MEM treatment also affects the phosphorylation of tau for mice without CRS stimulation, mice without CRS were treated with MEM in doses of 5 and 10 mg/kg for 16 consecutive days; Western blotting showed that treatment with MEM in doses of 5 and 10 mg/kg for 16 consecutive days caused a tendency to increase the tau phosphorylation at the AT8 epitope but had no statistical significance. In addition, total tau (TAU5) levels showed no statistical significant change in mice which were treated with either doses of MEM for 16 consecutive days (Figures 2(f)–2(h)).

### 3.3. MEM Reverses the Upregulation of GSK3*β* and CDK5 Levels and the Downregulation of PP2A Activity Caused by 16-Day CRS

**Figure 3:**
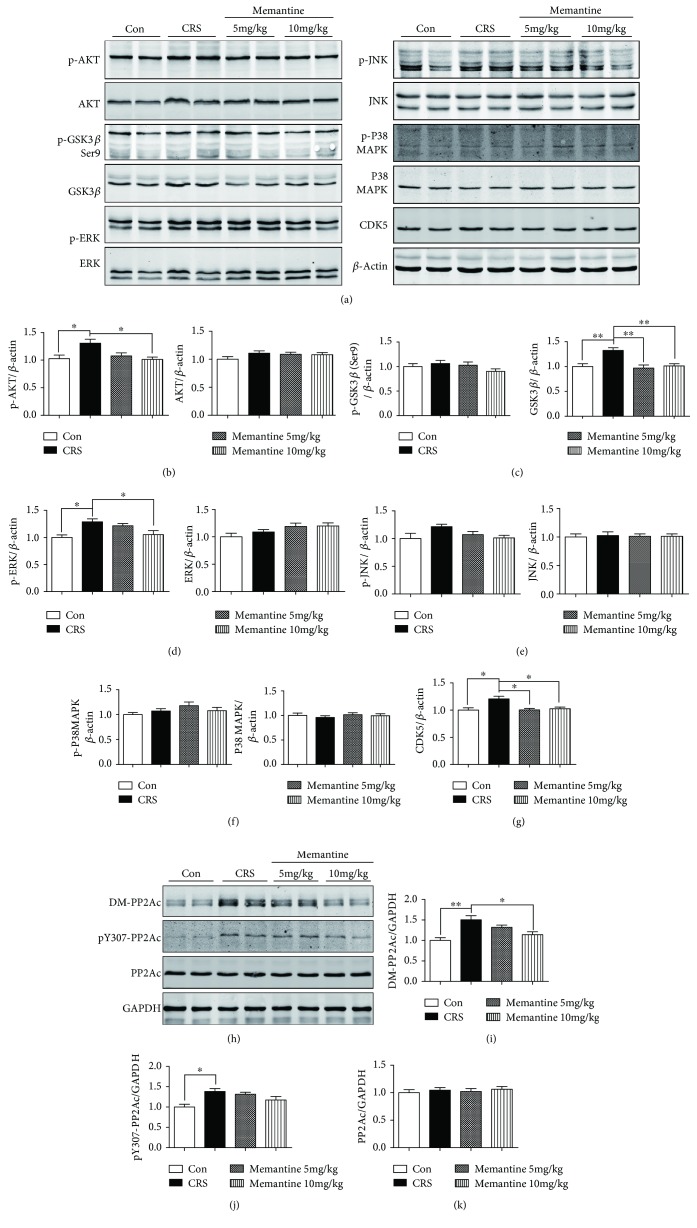
Effects of memantine on the changes in tau kinases and phosphatase induced by 16 days of chronic restraint stress (CRS). (a) Frontal cortex extracts from the four groups of mice were immunoblotted with antibodies against kinases including phosphorylated AKT (p-AKT (Ser473)), AKT, p-GSK3*β* (Ser9), GSK3*β*, p-ERK (Thr202/Tyr204), ERK, p-JNK (Thr183/Tyr185), JNK, p-P38 MAPK (Thr180/Tyr182), P38 MAPK, and CDK5; CRS increased the levels of p-AKT, p-ERK, CDK5, and GSK3*β*. Low-dosage memantine reduced CDK5 and GSK3*β* expression, whereas high-dosage memantine reduced the protein levels of p-AKT, p-ERK, CDK5, and GSK3*β*. (b–g) Densitometric quantification of the bands in (a). (h) Western blotting analysis showed that 16-day CRS induced increased expression of DM-PP2Ac and pY307-PP2Ac. Memantine partially reversed the changes of DM-PP2Ac and pY307-PP2Ac. (i–k) Densitometric quantification of the bands in (h). The results are shown as means ± SEM (*n* = 4 in each group); *β*-actin and GAPDH were used as loading controls. ^∗^*p* < 0.05; ^∗∗^*p* < 0.01. Con: control.

As mentioned above, MEM ameliorated the increase in tau phosphorylation induced in the brain by 16-day CRS. Therefore, we analyzed the levels of tau kinases and their activated forms (Figures 3(a)–3(g)). Western blotting showed that 16-day CRS significantly increased the levels of GSK3*β* (*p* < 0.01, Figures 3(a) and 3(c)) and CDK5 (*p* < 0.05, Figures 3(a) and 3(g)) and also upregulated the levels of p-AKT-Ser473 (Figures 3(a) and 3(b)) and p-ERK-Thr202/Tyr204 (Figures 3(a) and 3(d)), which are active forms of these kinases. MEM treatment significantly suppressed the CRS-induced upregulation of CDK5 (Figures 3(a) and 3(g)), GSK3*β* (Figures 3(a) and 3(c)), p-AKT (Figures 3(a) and 3(b)), and p-ERK levels (Figures 3(a) and 3(d)) but had no significant effect on the other tau-phosphorylating kinases or their activated forms.

Moreover, we also detected the activity and expression of protein phosphatase 2A (PP2A), which is the major tau phosphatase, accounting for approximately 70% of tau dephosphorylation in the human brain [39]. PP2A activity is regulated by its methylation and phosphorylation at its C-terminus of the catalytic subunit (PP2Ac). PP2Ac demethylation at Leu309 (DM-PP2Ac) or phosphorylation at Tyr307 (pY307-PP2Ac) will reduce the activity of PP2A and facilitates the phosphorylation of tau [40, 41]. Results showed that 16-day CRS increased the levels of DM-PP2Ac (*p* < 0.01, Figures 3(h) and 3(i)) and pY307-PP2Ac (*p* < 0.05, Figures 3(h) and 3(j)). However, there was no significant effect on the expression level of PP2Ac. MEM (10 mg/kg) treatment significantly suppressed the CRS-induced upregulation of DM-PP2Ac (*p* < 0.01, Figures 3(h) and 3(i)) and had an obvious tendency to reduce the level of pY307-PP2Ac (Figures 3(h) and 3(j)) but had no significant effect on the expression level of PP2Ac (Figures 3(h) and 3(k)). Meanwhile, MEM (5 mg/kg) treatment also caused a decrease tendency of DM-PP2Ac and pY307-PP2Ac, although without significance. These results suggested that CRS could induce the downregulation of PP2A activity and MEM treatment could reverse this abnormality.

### 3.4. MEM Treatment Reduces NMDA Receptor Expression Induced by 16-Day CRS

NMDAR subunits are encoded by three gene families, GluN1, GluN2 (A-D), and GluN3 (A/B) [42]. The most widely expressed NMDARs contain the obligatory GluN1 subunit combined with GluN2A, GluN2B, or both [43]. GluN2A- and GluN2B-containing NMDARs have differential roles in central nervous system functions [44]. They also might play different roles in the regulation of tau phosphorylation [45, 46]. To explore the mechanism of the effect of MEM on tau phosphorylation induced by 16-day CRS, we assessed the expression of GluN2A and GluN2B after CRS with or without MEM treatment.

**Figure 4:**
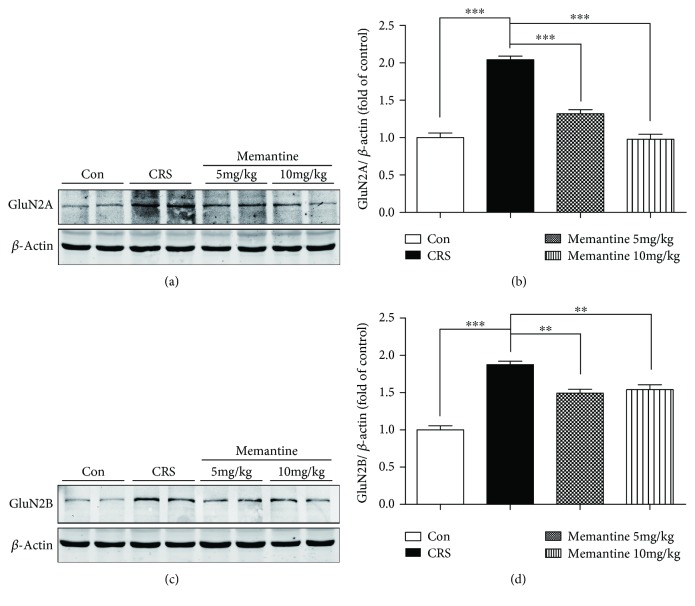
Effects of memantine on N-methyl-D-aspartate receptor (NMDAR) expression in the frontal cortex of mice subjected to 16 consecutive days of chronic restraint stress (CRS). (a, c) Western blotting of frontal cortex extracts from the four groups of mice showed that GluN2A and GluN2B expression levels were significantly elevated in the CRS group. Memantine reversed the CRS-induced upregulation of NMDAR expression at both dosages. (b, d) Densitometric quantification of the bands in (a). The results are shown as means ± SEM (*n* = 4 in each group); *β*-actin was used as a loading control. ^∗∗^*p* < 0.01; ^∗∗∗^*p* < 0.001. Con: control.

Western blot analysis showed that CRS significantly increased the expression of NMDARs containing GluN2A (*p* < 0.001, Figures 4(a) and 4(b)) and GluN2B (*p* < 0.001, Figures 4(c) and 4(d)) in the frontal cortex of mice subjected to 16-day CRS. However, treatment with either 5 mg/kg or 10 mg/kg MEM significantly suppressed the CRS-induced upregulation of GluN2A and GluN2B expression (Figure 4).

### 3.5. MEM Enhances Tau Phosphorylation in the Frontal Cortex of Mice Subjected to 28-Day CRS

**Figure 5:**
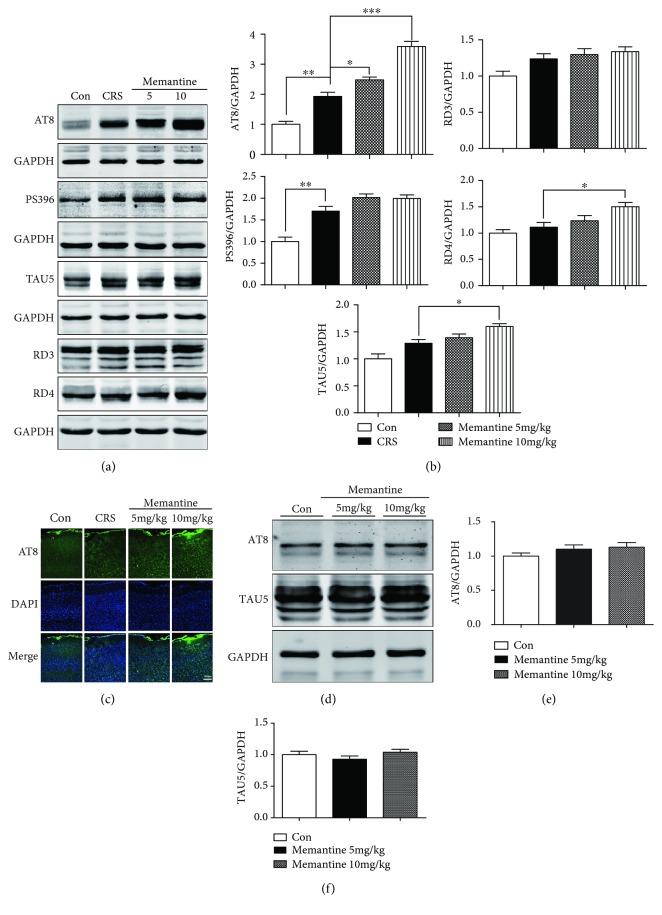
Memantine enhances tau phosphorylation after 28-day chronic restraint stress (CRS) in the mouse frontal cortex. (a) Results of Western blotting showed that memantine enhances tau phosphorylation at the AT8 site for both doses. Furthermore, the expression of the 3R tau isoform (RD3), 4R tau isoform (RD4), P-tau-PS396, and TAU5 was also detected among the four groups of mice. (b) Densitometric quantification of the bands in (a). (c) Representative areas of AT8-positive immunofluorescent staining in cortices from the four groups of mice. (d) Western blotting analyzed the effects of memantine administration with doses of 5 and 10 mg/kg for 28 days on tau phosphorylation and expression in frontal cortex of mice without CRS. (e, f) Densitometric quantification of the bands in (d). The results are shown as means ± SEM (*n* = 4 in each group). GAPDH was used as a loading control. ^∗^*p* < 0.05; ^∗∗^*p* < 0.01; ^∗∗∗^*p* < 0.001. Con: control.

MEM has been shown to inhibit tau phosphorylation in AD model mice [47]. As described above, MEM can reduce the phosphorylation of tau induced by CRS for 16 consecutive days. Whether MEM has the similar effects on the regulation of tau phosphorylation induced by CRS of varying duration still requires further research. In this study, results showed increased tau phosphorylation in the frontal cortex of mice subjected to 28-day CRS. Surprisingly, we found that MEM treatment enhanced the increase in AT8-reactive P-tau induced in the frontal cortex by 28-day CRS (Figures 5(a) and 5(b)). Immunofluorescent staining confirmed the phosphorylation-promoting effect of MEM (Figure 5(c)). In addition, an increase level of P-tau labeled by the PS396 antibody was also presented in the frontal cortex after 28-day CRS (*p* < 0.01, Figures 5(a) and 5(b)). In contrast, MEM treatment also caused an increase tendency of P-tau-PS396, although without significance (*p* > 0.05, Figures 5(a) and 5(b)).

Moreover, MEM (10 mg/kg) treatment elevated the total tau protein level (*p* < 0.05, Figures 5(a) and 5(b)) as assessed with a TAU5 antibody. Tau gene exon 10 encodes the second microtubule-binding repeat. In the adult brain, alternative splicing of exon 10 generates tau isoforms containing three or four microtubule-binding repeats, named 3R-tau and 4R-tau, respectively. Imbalanced expression of 3R- and 4R-tau is known to be sufficient to induce neurofibrillary degeneration in tauopathies [48]. Thus, we also examined the 3R- and 4R-tau levels, with RD3 and RD4 antibodies. 4R-tau rather than 3R-tau was associated with increase of tau due to the MEM (10 mg/kg) treatment (*p* < 0.05, Figures 5(a) and 5(b)). These results suggested that MEM treatment enhances tau expression and phosphorylation in mice subjected to CRS.

Also, we investigated the effects of MEM in doses of 5 and 10 mg/kg for 28 days in mice without CRS. We found that MEM treatment caused an increasing trend of the AT8-reactive P-tau level in the frontal cortex of mice without CRS but without statistical significance (Figures 5(d) and 5(e)). Both doses of MEM treatment had no obvious influence on the total tau level (Figures 5(d) and 5(f)).

### 3.6. MEM Enhances Tau Phosphorylation in Other Parts of the Cerebral Cortex of Mice Subjected to 28-Day CRS

**Figure 6:**
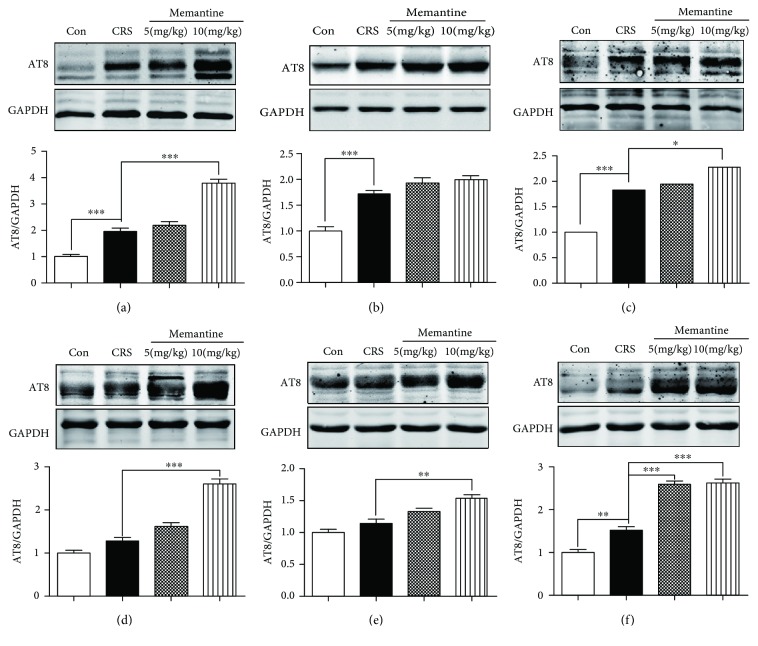
Memantine enhances tau phosphorylation in hippocampus and several subregions of the cerebral cortex of mice during 28-day chronic restraint stress (CRS). Western blotting detected the AT8 antibody-labeled phosphorylated tau in (a) hippocampus, (b) anterior cingulate cortex, (c) posterior cingulate cortex, (d) parietal cortex, (e) occipital cortex, and (f) entorhinal cortex, and the quantitative analysis normalized against GAPDH. The results are shown as means ± SEM (*n* = 4 in each group). ^∗^*p* < 0.05; ^∗∗^*p* < 0.01; ^∗∗∗^*p* < 0.001. Con: control.

The increase in AT8-labeled phosphorylated tau levels was present not only in the frontal cortex (Figures 5(a) and 5(b)) but also in the hippocampus (*p* < 0.001, Figure 6(a)) and in other subregions of the cerebral cortex, such as the anterior cingulate cortex (*p* < 0.001, Figure 6(b)), posterior cingulate cortex (*p* < 0.001, Figure 6(c)), and entorhinal cortex (*p* < 0.01, Figure 6(f)). To evaluate whether the effect of MEM was specific for the frontal cortex, we also analyzed other brain structures. Results showed that low-dose MEM (5 mg/kg) promoted tau phosphorylation in the entorhinal cortex (*p* < 0.001, Figure 6(f)), while high-dose MEM (10 mg/kg) stimulated tau phosphorylation in the hippocampus (*p* < 0.001, Figure 6(a)), posterior cingulate cortex (*p* < 0.05, Figure 6(c)), parietal cortex (*p* < 0.001, Figure 6(d)), occipital cortex (*p* < 0.01, Figure 6(e)), and entorhinal cortex (*p* < 0.001, Figure 6(f)) for mice subjected to 28-day CRS.

### 3.7. Effects of MEM on Changes in Tau Kinases and Phosphatase Induced by 28-Day CRS

**Figure 7:**
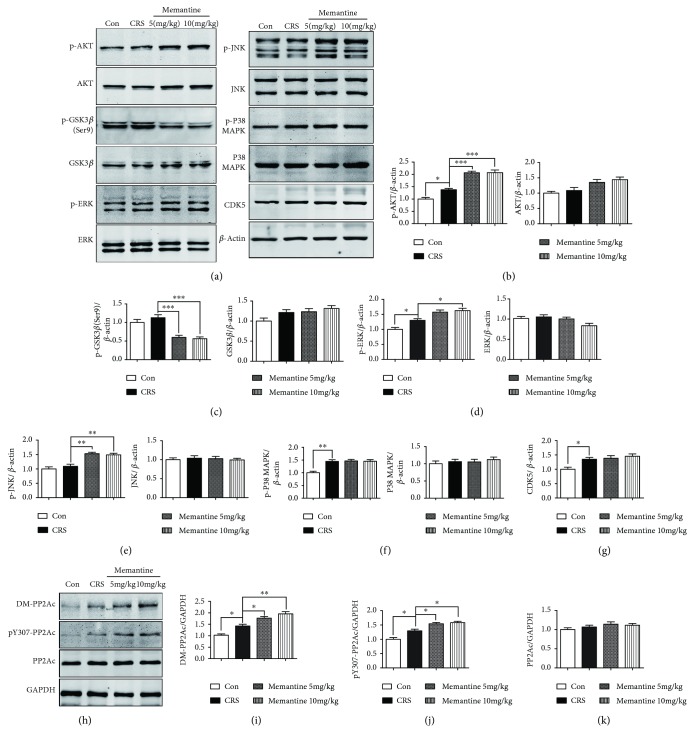
Modulation of tau kinases and phosphatase by memantine after 28 consecutive days of chronic restraint stress (CRS). (a) Frontal cortex lysates from the four mouse groups were immunoblotted with antibodies against p-AKT (Ser473), AKT, p-GSK3*β* (Ser9), GSK3*β*, p-ERK (Thr202/Tyr204), ERK, p-JNK (Thr183/Tyr185), JNK, p-P38 MAPK (Thr180/Tyr182), P38 MAPK, and CDK5; memantine increased the levels of p-AKT, p-ERK, and p-JNK, while reducing those of p-GSK3*β*. (b–g) Densitometric quantification of the bands in (a). (h) Western blotting showed that 28-day CRS induced increase of DM-PP2Ac and pY307-PP2Ac, and memantine enhanced the levels of DM-PP2Ac and pY307-PP2Ac. (i–k) Densitometric quantification of the bands in (h). The results are shown as means ± SEM (*n* = 4 in each group); *β*-actin and GAPDH were used as loading controls. ^∗^*p* < 0.05; ^∗∗^*p* < 0.01; ^∗∗∗^*p* < 0.001. Con: control.

To investigate the potential mechanism of the effects of MEM on tau phosphorylation induced by 28-day CRS, we examined the levels of tau kinases, including CDK5, GSK-3*β*, ERK, JNK, and P38 MAPK and their phosphorylated forms (Figures 7(a)–7(g)). Twenty-eight days of CRS significantly stimulated the expression of CDK5 (*p* < 0.05, Figures 7(a) and 7(g)) and induced a trend-level increase in GSK3*β* expression (Figures 7(a) and 7(c)). Significant upregulation was also observed in the levels of p-AKT-Ser473 (Figures 7(a) and 7(b)), p-ERK-Thr202/Tyr204 (Figures 7(a) and 7(d)), and p-P38 MAPK-Thr180/Tyr182 (Figures 7(a) and 7(f)), which are active forms of these kinases. With MEM-treated CRS mice, CDK5 expression was sustained at a high level similar to that in CRS-only mice. MEM (10 mg/kg) significantly reduced the levels of GSK3*β* phosphorylated at Ser9, the inactive form of GSK3*β* (*p* < 0.001, Figures 7(a) and 7(c)), and increased the levels of p-AKT (*p* < 0.001, Figures 7(a) and 7(b)), p-JNK (*p* < 0.01, Figures 7(a) and 7(e)), and p-ERK (*p* < 0.05, Figures 7(a) and 7(d)) compared to those in CRS-only mice. Because CDK5 and GSK3*β* are directly involved in tau phosphorylation, the significant decrease in inactive GSK3*β* suggests that GSK3*β* activation may be involved in the MEM-induced increase in P-tau levels during the 28-day CRS.

In addition, we found the increased of DM-PP2Ac (*p* < 0.05, Figures 7(h) and 7(i)) and pY307-PP2Ac (*p* < 0.05, Figures 7(h) and 7(j)) levels induced by 28-day CRS. MEM (5 mg/kg) treatment increased the levels of DM-PP2Ac (*p* < 0.05) and pY307-PP2Ac (*p* < 0.05). MEM (10 mg/kg) treatment could significantly further upregulate the levels of DM-PP2Ac (*p* < 0.01) and pY307-PP2Ac (*p* < 0.05). Neither CRS nor CRS with MEM treatment had a statistically significant impact on the expression levels of PP2Ac. These data suggest that the inactivation of PP2A is involved in the increase of P-tau and the exacerbating effect of MEM treatment on tau phosphorylation during the 28 days of CRS.

### 3.8. MEM Modulates Changes in Chaperone Protein Expression Induced by 28-Day CRS

**Figure 8:**
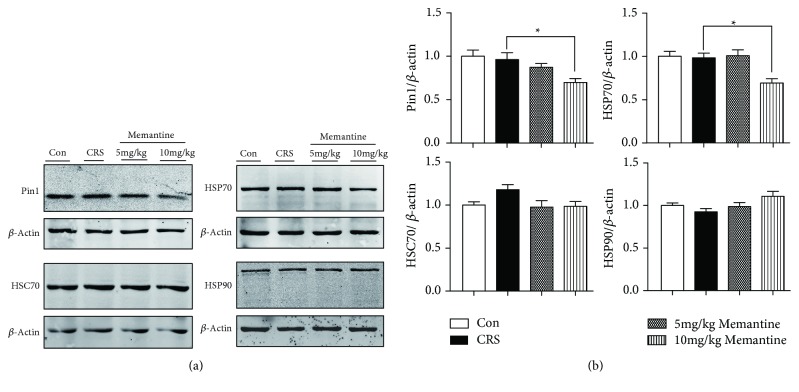
The effect of memantine on the expression of chaperone proteins in mice following 28 consecutive days of chronic restraint stress (CRS). (a) Frontal cortex lysates from the four groups were immunoblotted with antibodies against Pin1 and heat shock proteins HSP70, HSC70, and HSP90. (b) Densitometric quantification of the bands in (a). The results are shown as means ± SEM (*n* = 4 in each group); *β*-actin was used as a loading control. ^∗^*p* < 0.05. Con: control.

To further explore the mechanism underlying increased tau phosphorylation in CRS mice treated with MEM, we also analyzed proteins involved in the regulation of tau degradation and aggregation, including Pin1 and molecular chaperones such as heat shock proteins HSP70, HSC70, and HSP90. Twenty-eight days of CRS had no significant influence on the expression of Pin1, HSC70, HSP70, and HSP90. The higher MEM dose (10 mg/kg) significantly downregulated the expression of Pin1 (*p* < 0.05) and HSP70 (*p* < 0.05) in 28-day CRS mice (Figures 8(a) and 8(b)).

### 3.9. MEM Modulates the Induction of NMDAR Expression by 28-Day CRS

**Figure 9:**
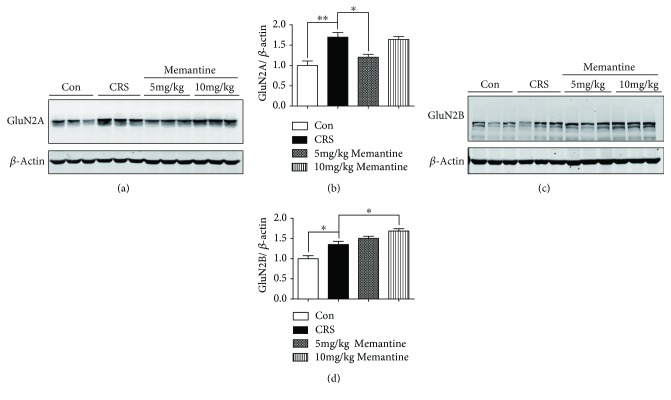
Memantine regulates N-methyl-D-aspartate receptor (NMDAR) expression in mice subjected to 28 consecutive days of chronic restraint stress (CRS). (a, c) Western blotting of the frontal cortex extracts showed that CRS increased the protein expression levels of the NMDAR subunits GluN2A and GluN2B. In contrast to the CRS group, low-dosage memantine partially suppressed GluN2A expression while tending to increase the expression of GluN2B. High-dosage memantine increased the protein expression levels of GluN2B; however, it did not have a statistically significant effect on GluN2A levels. (b, d) Densitometric quantification of the bands in (a) and (c). The results are shown as means ± SEM (*n* = 4 in each group); *β*-actin was used as a loading control. ^∗^*p* < 0.05; ^∗∗^*p* < 0.01. Con: control.

Western blot analysis showed a significant increase in GluN2A (*p* < 0.01, Figures 9(a) and 9(b)) and GluN2B (*p* < 0.05, Figures 9(c) and 9(d)) expression induced by 28-day CRS. Treatment with 5 mg/kg MEM suppressed the upregulation of GluN2A (*p* < 0.05) but not GluN2B expression. However, treatment with 10 mg/kg MEM did not significantly affect the GluN2A level in CRS-only mice, whereas GluN2B expression was further enhanced (*p* < 0.05). Because the overactivation of GluN2B-containing NMDARs was reported to result in the accumulation of hyperphosphorylated tau in the hippocampus [32], our results indicate a potential link between GluN2B-containing NMDARs and tau phosphorylation in the frontal cortex of mice exposed to 28-day CRS with MEM treatment.

## 4. Discussion

### 4.1. Anxiolytic Effect of MEM

The glutamate neurotransmitter system has been considered to be involved in psychiatric disease. In this study, EPM testing showed that MEM had anxiolytic effects in mice subjected to both 14- and 27-day CRS, consistent with its effect in a chronic mild unexpected stress model [49]. However, studies on the potential of MEM to alleviate anxiety have produced mixed results, with some studies reporting an anxiolytic-like effect [49, 50] and others finding MEM to be ineffective as an anxiolytic [51, 52]. The discrepancy may be attributable to different efficacies of MEM in different animal models. Previous studies have implicated AKT-GSK3*β* signaling in anxiety-like behavior after alcohol withdrawal [53]. In our study, CRS-induced anxiety-like behavior was accompanied by an upregulation of p-AKT levels following both short and long CRS regimens. MEM treatment attenuated the upregulation of p-AKT induced by 16-day CRS; however, it enhanced AKT phosphorylation following 28-day CRS. Therefore, we propose that AKT activation in the frontal cortex might not be a determinant of CRS-induced anxiety-like behavior. In addition, mice gained body weight gradually in the control group, while the body weights of mice exposed to chronic stress fluctuated during the course of the experiment, albeit remaining lower than that of the control mice. However, MEM-treated animals had a similar mean body weight to that of stressed animals. These results suggest that different mechanisms may be involved in the control of anxiety and body weight in response to CRS [54].

### 4.2. CRS, NMDARs, and Tau Phosphorylation

Data from our group and others have demonstrated that exposure to acute or chronic stress elevates tau phosphorylation levels in the brain [20, 22, 55]. Moreover, stress also enhanced tau phosphorylation in a transgenic mouse model of tauopathy [17]. The results of these studies are consistent with epidemiological researches [6, 7], suggesting that stress may be involved in AD pathogenesis. Several studies have indicated that the corticotropin-releasing factor (CRF)/CRF receptor 1 (CRFR1) signaling mediates stress-induced tau phosphorylation [17, 18, 34, 56]. However, another study reported that CRF attenuated stress-induced tau neuropathology, particularly in conditions of chronic stress [57]. In addition, stress was reported to induce an increase in glutamate release [58], and the noncompetitive NMDAR antagonist MK-801 attenuated tau phosphorylation in a chronic noise model (4 h/day for 14 consecutive days) [38]. These findings not only suggest a possible role of glutamate in the mechanisms underlying molecular and cellular alterations in the brain caused by stress but also imply that the regulation of glutamatergic function may attenuate the stress-induced pathological changes. However, whether the NMDAR antagonist MEM can minimize CRS-induced tau phosphorylation has not been previously evaluated. Therefore, in this study, we examined the effect of the NMDAR antagonist MEM on tau phosphorylation induced by CRS.

MEM has been used in AD treatment. Both clinical and preclinical studies have shown that MEM, at doses producing a steady-state plasma level of 0.5-1 *μ*M, is well tolerated and improves cognition in patients with AD [50]. Acute i.p. doses of 5.0 mg/kg of MEM produce a plasma *C*_max_ of approximately 1 *μ*M in rodents [59]. Therefore, the doses of 5.0 and 10 mg/kg of MEM were used in this study. MEM treatment significantly attenuated tau hyperphosphorylation in the frontal cortex of mice subjected to 16-day CRS, with the reduction being more pronounced at the dose of 10 mg/kg. Considering these findings, it is likely that Glu-NMDAR signaling may be involved in certain aspects of CRS-induced tau hyperphosphorylation.

### 4.3. Mechanisms of the Inhibitory Effect of MEM on CRS-Induced Tau Phosphorylation

The state of tau phosphorylation is related to a complex regulatory network of protein kinases and phosphatases, among which GSK3*β* and CDK5 are the most prominent tau kinases [60] and PP2A is the most important phosphatase for tau dephosphorylation. Our results showed that MEM could suppress the upregulation of GSK3*β* and CDK5 expression and minimize the downregulation of PP2A activity induced by 16-day CRS, which was mirrored in the downregulation of CRS-induced tau phosphorylation at the AT8 epitope.

The studies have shown that AKT could regulate the activity of GSK-3*β* via phosphorylation at Ser9 by Cross et al. [61], while another line study showed that AKT can prevent AMPK-induced tau dephosphorylation [62]. Thus, we also investigated the levels and phosphorylation state of this upstream GSK-3*β* regulator, the AKT. We found that 16-day CRS induced an increase in AKT phosphorylated at Ser473, the active form of AKT, which could be minimized by MEM. It should be noted that the activation of AKT did not result in a corresponding increase in GSK-3*β* Ser9 phosphorylation, which did not show a significant difference between the CRS and CRS + MEM groups. These results suggest that AKT is not the sole upstream regulator of GSK-3*β*.

In addition, ERKs and JNKs, which are the major brain MAPKs, play important roles in neuronal differentiation and development as well as in tau phosphorylation [60, 63–66]. In the present study, the levels of p-ERKs and p-JNKs, the active forms of these kinases, also increased or tended to increase, after CRS stimulation, and the increases could be suppressed by MEM treatment. These results provide evidence for a role of NMDARs in CRS-induced AD-like neuropathological tau alterations. The findings also raise the possibility that noncompetitive NMDAR antagonists may attenuate some of the pathophysiological damage following CRS exposure.

### 4.4. Mechanisms of the Stimulatory Effect of MEM on Tau Phosphorylation Induced by 28-Day CRS

We found that MEM enhanced tau phosphorylation in the frontal cortex and other brain regions, including the hippocampus, following 28-day CRS. In this condition, MEM treatment upregulated the p-JNK, p-AKT, and p-ERK levels compared to those in CRS-only mice. MEM treatment also significantly suppressed the phosphorylation of GSK3*β* at Ser9, resulting in GSK3*β* activation, whereas the CDK5 level remained similar to that in CRS-only mice. In addition, MEM treatment significantly increased the levels of PP2A phosphorylated at tyrosine 307 (Y307) and PP2A demethylation at Leu309 relative to CRS-only mice. Therefore, treatment with MEM for mice subjected to 28-day CRS may activate kinases, GSK3*β* in particular, and inactivate phosphatase PP2A, contributing to MEM enhanced CRS-induced tau phosphorylation. In addition, treating two doses of MEM without CRS caused an increase in AT8-reactive P-tau levels in the frontal cortex of mice but without statistical significance.

Moreover, the effects of MEM treatment on the expression of chaperone proteins might be also involved in the increased tau phosphorylation. Thus, we found the downregulation of Pin1 and HSP70 expression to correlate with increased tau phosphorylation. Our results are consistent with reports showing that Pin1 deficiency promotes tau phosphorylation, pathological changes, and neurodegeneration [67–69] and that HSP70 may be involved in the prevention of abnormal tau aggregation [70]. Regarding MEM side effects, a report showed that MEM potentiated, rather than prevented, stress-induced reduction in cell proliferation and failed to modify the levels of hormones involved in the hypothalamic pituitary adrenal (HPA) axis [71]. However, Creeley et al. reported that MEM was neuroprotective only at doses producing intolerable side effects [72].

### 4.5. NMDAR Regulation of Tau Phosphorylation

Excitotoxicity-induced neuronal death or injury through NMDAR channels is known to be involved in both acute neuronal damage and chronic neurodegenerative diseases [73]. However, *in vitro* studies of the effects of glutamate on tau phosphorylation have yielded contradictory results. Some studies found NMDAR activation to induce tau phosphorylation [74], while others reported that glutamate treatment led to rapid dephosphorylation and proteolysis of tau [75–77]; NMDA treatment also caused a decrease in P-tau levels [78].

Recent studies also have shown that the overactivation of GluN2B-containing NMDARs leads to the accumulation of hyperphosphorylated tau [45, 46]. Another line studies indicated that the stimulation of synaptic NMDARs induces prosurvival events, whereas the activation of extrasynaptic NMDARs leads to excitotoxic death [79], although the synaptic receptors participate prominently in hypoxic excitotoxicity [80]. It is generally believed that GluN2B exists at extrasynaptic sites more abundantly than GluN2A, which are preferentially found at synapses [81]. It was found that the stimulation of extrasynaptic NMDARs causes the upregulation of the tau level, which was accompanied with a higher level of P-tau [82]. Considering that stress could increase extracellular glutamate levels [25, 26], the increase of the tau level and phosphorylated tau after CRS might be related to the activation of GluN2B containing NMDARs and possibly the extrasynaptic NMDAR activation.

The analysis of the effects of MEM on recombinant rat NMDA receptors expressed in HEK 293 cells showed that MEM blocked glutamate-mediated currents in a concentration-dependent manner in GluN1a/GluN2A- and GluN1a/GluN2B-transfected cells with IC50 values (at −70 mV) of 0.93 ± 0.15 *μ*M and 0.82 ± 0.12 *μ*M, respectively [83]. The IC50 value for GluN2B lower than GluN2A is corresponding to that MEM preferentially inhibits extrasynaptic NMDA receptors in hippocampal neurons [84]; moreover, MEM also could selectively block extrasynaptic NMDA receptors in rat substantia nigra dopamine neurons, which contain GluN2C/2D subunits [85]. Hence, MEM treatment-suppressed tau phosphorylation in 16-day CRS experiments might be via compromising the activity of GluN2B or extrasynaptic NMDARs. However, it is still not clear whether NMDARs are involved in the deteriorative effect of MEM on 28-day CRS caused by P-tau. Therefore, in this study, we explored the underlying mechanism of the stimulatory effect of MEM on tau phosphorylation by examining the expression of different NMDARs. Our results showed that CRS caused an increase in the levels of NMDARs containing GluN2A and GluN2B, which is in accordance with the previous studies [86], while MEM treatment suppressed the expression of both the GluN2A and GluN2B NMDAR subunits after 16-day CRS. In contrast, treatment with 5 mg/kg MEM did not suppress GluN2B upregulation following 28-day CRS and treatment with 10 mg/kg MEM even induced the upregulation of GluN2B expression compared to that in CRS-only mice. Li et al. found increased levels of p-ERK in the cerebral cortices of GluN2B transgenic mice [87], which is consistent with our results showing p-ERK upregulation to be accompanied by increasing GluN2B expression in MEM-treated CRS mice. The deregulation of GluN2B expression might cause abnormal distribution of the NMDA receptor on the extrasynaptic region, which then subsequently contributes to the MEM-induced enhancement of tau phosphorylation for mice subjected to 28-day CRS. However, the mechanism of MEM regulating GluN(2A/2B) expression remains to be studied further.

## 5. Conclusions

To our knowledge, the relationship between NMDAR signaling and tau phosphorylation after CRS has not been examined. In the present study, we demonstrated that MEM significantly attenuated aberrant tau phosphorylation in the frontal cortex following CRS exposure for 16 consecutive days, while enhancing the tau phosphorylation abnormality after 28-day CRS. The CRS duration-dependent effects of MEM on tau phosphorylation were correlated with the changes in tau kinases and phosphatase. The underlying mechanism of the stimulatory effect of MEM on CRS-induced tau hyperphosphorylation might be related to the upregulation of GluN2B expression. The differential effects of MEM on pathological tau changes have important implications for its clinical applications, including the need for refining the dosage and regimen of any potential MEM-based therapy. Future studies are also required to determine whether MEM may also deregulate the expression of GluN2B and promote tau phosphorylation when used for the treatment of other neurological disorders.

## Data Availability

The data used to support the findings of this study are included within the article.
